# Supplementary material for: Selective cobalt and nickel electrodeposition for lithium-ion battery recycling through integrated electrolyte and interface control
Source: Nat Commun. 2021 Nov 12;12:6554. doi: 10.1038/s41467-021-26814-7 (PMC8590046; doi:10.1038/s41467-021-26814-7)
Supplement: Supplementary file 1 — Supplementary Information [file 41467_2021_26814_MOESM1_ESM.pdf]

Supplementary Information

Selective Cobalt and Nickel Electrodeposition for Lithium-ion Battery Recycling through Integrated Electrolyte and Interface Control

Kwiyong Kim[a], Darien Raymond[a], Riccardo Candeago[a], Xiao Su[a]*

[a] Department of Chemical and Biomolecular Engineering, University of Illinois at Urbana-Champaign, Urbana, Illinois 61801 (United States). E-mail: x2su@illinois.edu

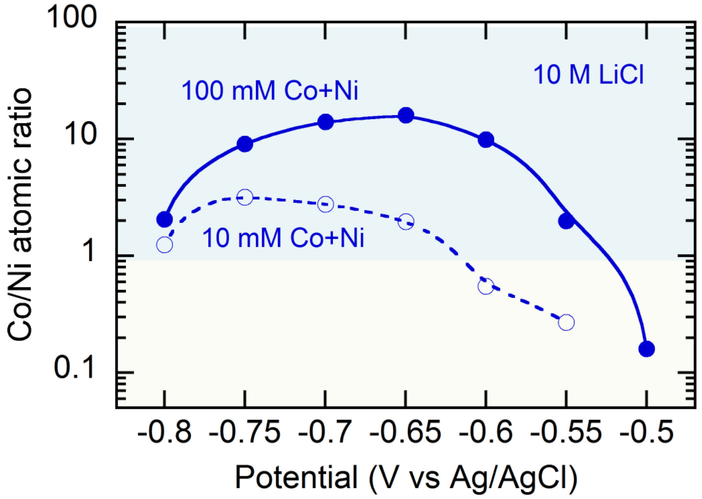

Supplementary Figure 1. Surface Co/Ni ratios on the electrodeposits formed in the binary mixture of 10 mM Co(II)+Ni(II) and 100 mM Co(II)+Ni(II) in 10 M LiCl. The substrate was copper foil. The enhancement in cobalt selectivity with the increase in bulk concentrations is in agreement with the fact that cobalt deposition is mass transfer-controlled during anomalous electrodeposition[1]. On the other hand, the increase in the nickel content (the decrease in cobalt content) at highly negative potential (*e.g.*, at -0.8 V vs Ag/AgCl) implies that nickel deposition is activation-controlled[1, 2].

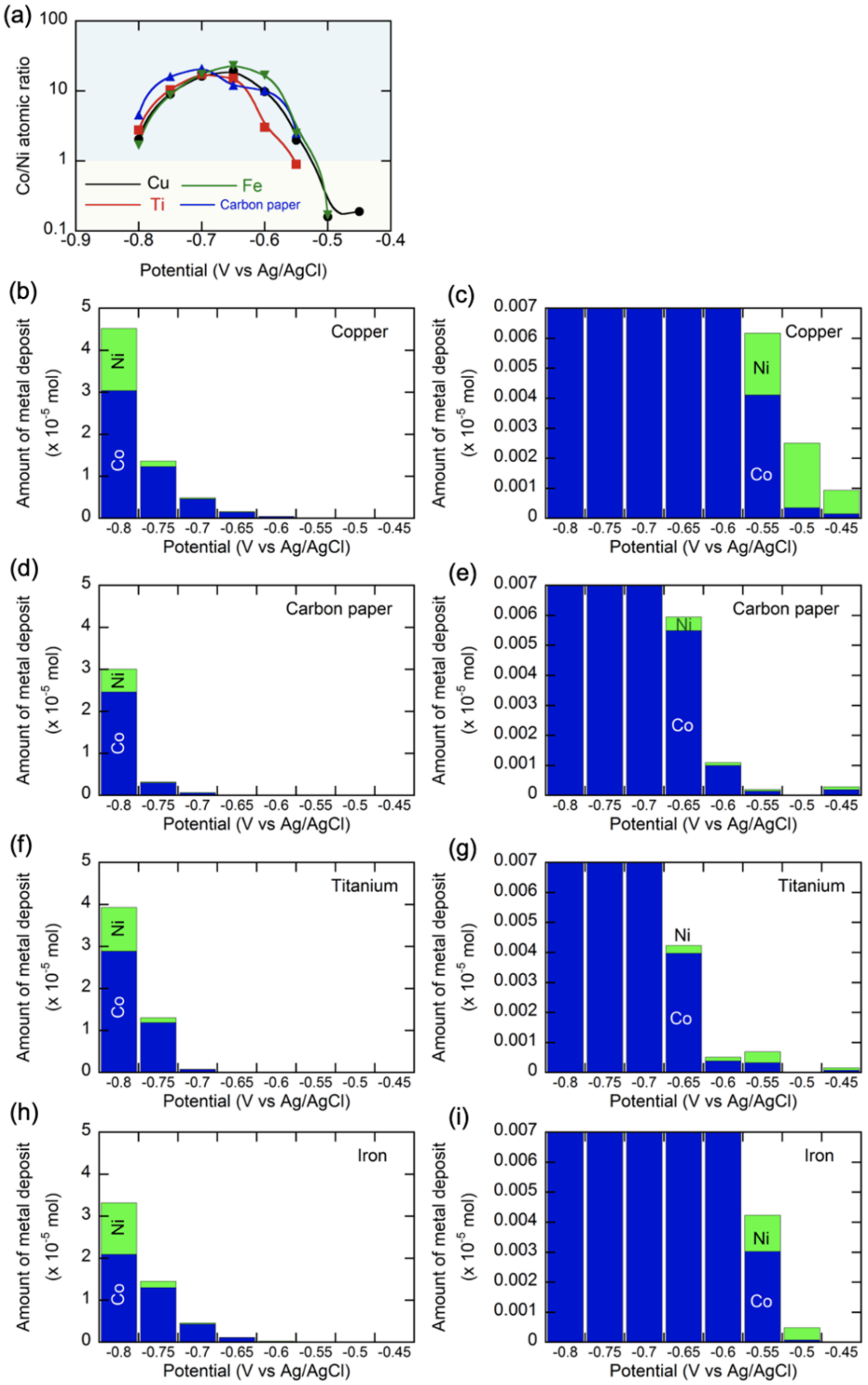

Supplementary Figure 2. (a) Effect of applied potential on the surface Co/Ni ratios on the electrodeposits formed in the binary mixture of 100 mM Co(II)+Ni(II) in 10 M LiCl using various substrates as a working electrode. (b-i) Effect of applied potential on the actual amount of cobalt and nickel electrodeposited on various substrates: (b, c) copper, (d, e) carbon paper, (f, g) titanium, (h, i) iron. (c), (e), (g), and (i) are the magnified view of (b), (d), (f), and (h), respectively.

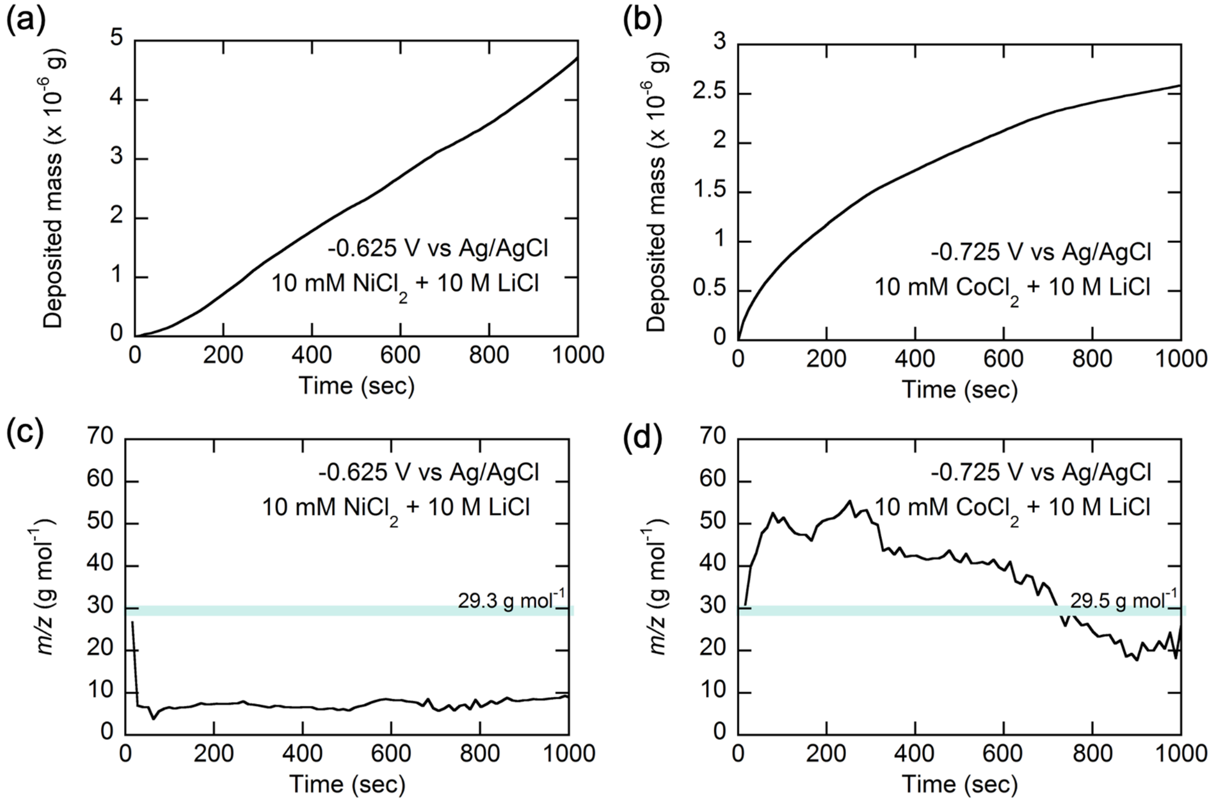

Supplementary Figure 3. (a) and (b): Increase in mass during the electrodeposition of (a) nickel in 10 mM Ni(II) + 10 M LiCl at -0.625 V vs Ag/AgCl and (b) cobalt in 10 mM Co(II) + 10 M LiCl at -0.725 V vs Ag/AgCl. (c) and (d): Specific mass change per number of electrons (*m/z*) during the electrodeposition of (c) nickel in 10 mM Ni(II) + 10 M LiCl at -0.625 V vs Ag/AgCl and (d) cobalt in 10 mM Co(II) + 10 M LiCl at -0.725 V vs Ag/AgCl.

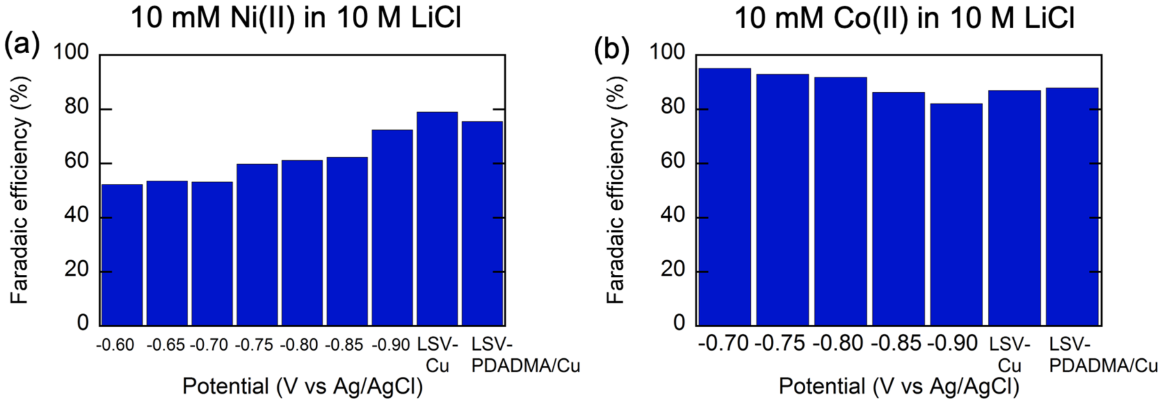

Supplementary Figure 4. (a) Faradaic efficiency of nickel electrodeposition in 10 mM Ni(II) + 10 M LiCl at various applied potentials in chronoamperometric operation or linear sweep voltammetry using Cu or PDADMA/Cu as a working electrode. (b) Faradaic efficiency of cobalt electrodeposition in 10 mM Co(II) + 10 M LiCl at various applied potentials in chronoamperometric operation or linear sweep voltammetry using Cu or PDADMA/Cu as a

working electrode. For the determination of Faradaic efficiency, each electrodeposit was digested and the molar amount of metals in digestion solution was quantified using ICP-OES.

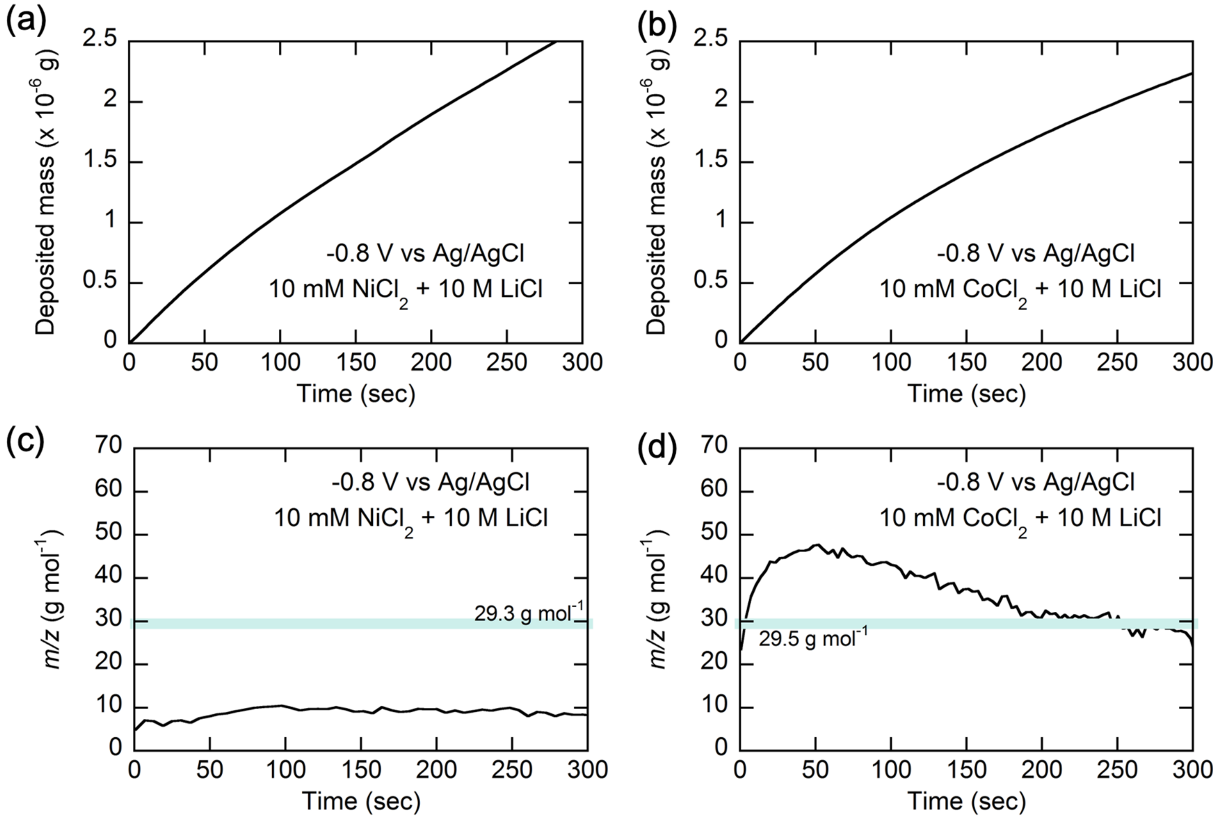

Supplementary Figure 5. (a) and (b): Increase in mass during the electrodeposition of (a) nickel in 10 mM Ni(II) + 10 M LiCl at -0.8 V vs Ag/AgCl and (b) cobalt in 10 mM Co(II) + 10 M LiCl at -0.8 V vs Ag/AgCl. (c) and (d): Specific mass change per number of electrons (*m/z*) during the electrodeposition of (c) nickel in 10 mM Ni(II) + 10 M LiCl at -0.8 V vs Ag/AgCl and (d) cobalt in 10 mM Co(II) + 10 M LiCl at -0.8 V vs Ag/AgCl.

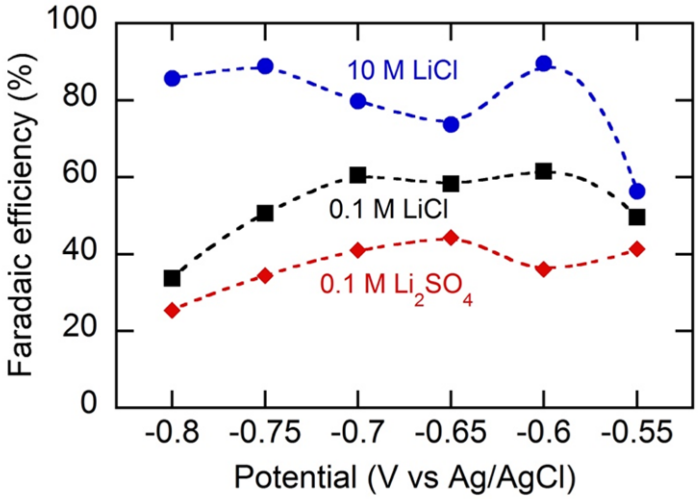

Supplementary Figure 6. Faradaic efficiency of cobalt and nickel electrodeposition in 10 mM Ni(II)+Co(II) in various background electrolytes of 0.1 M Li_2SO_4, 0.1 M LiCl, and 10 M LiCl. The working electrode was a pristine copper foil.

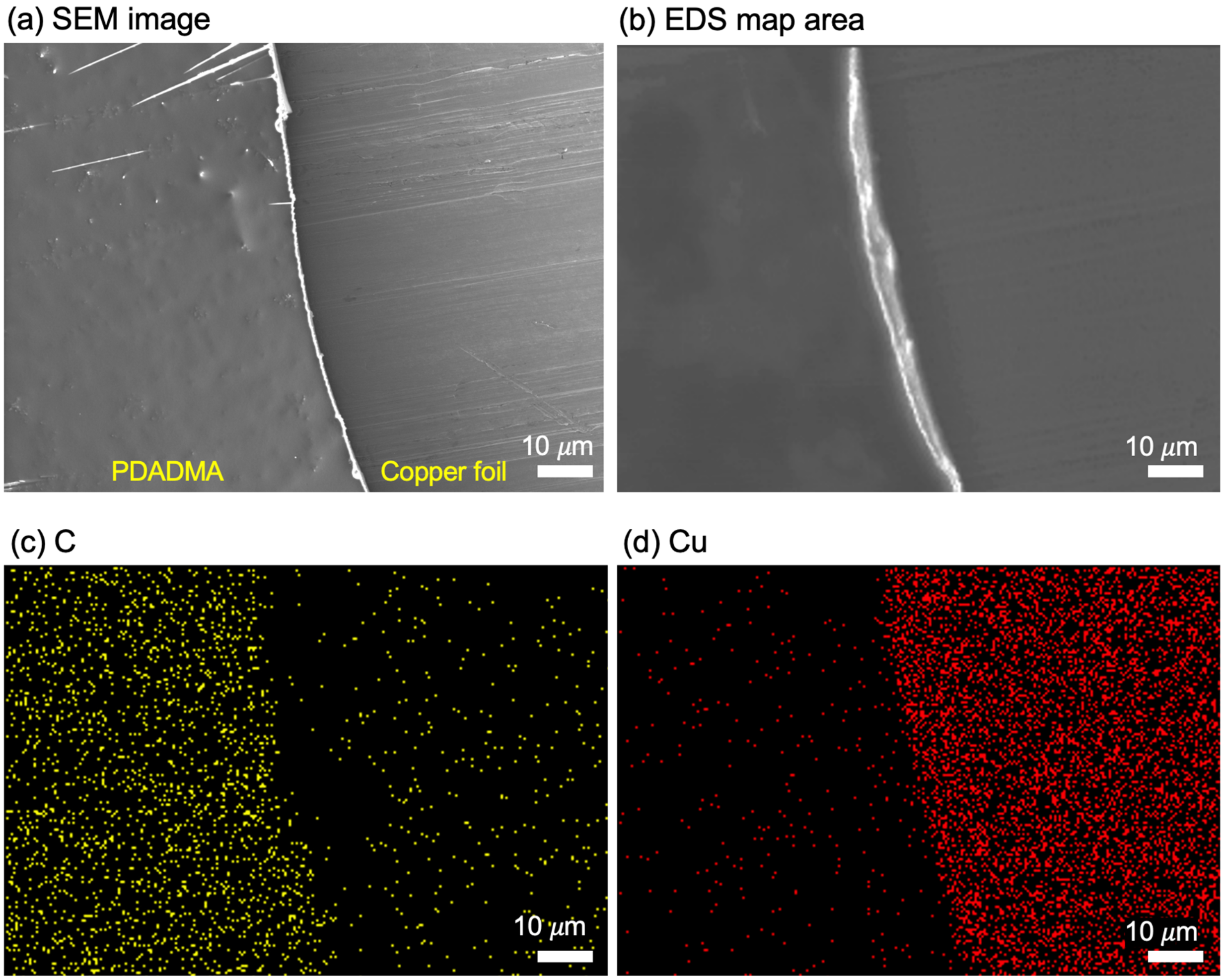

Supplementary Figure 7. Scanning electron microscopy (SEM) images and EDS mapping of carbon and copper on a PDADMA/Cu electrode (PDADMA loading: 0.75 mg cm^{-2}). All scale bars are 10 μm.

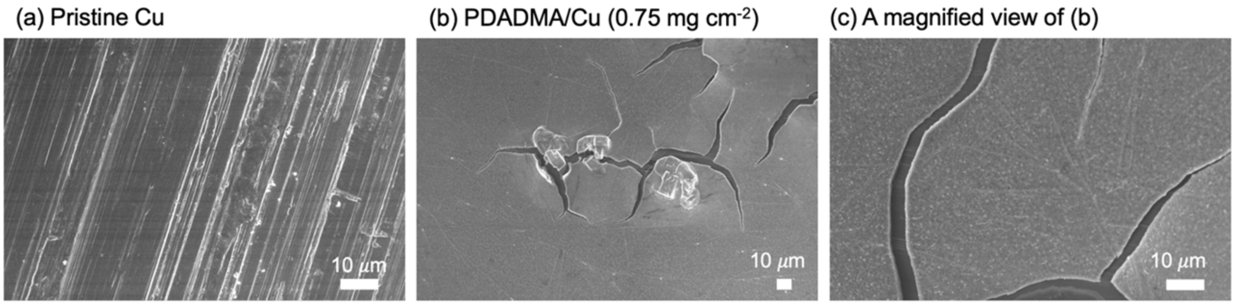

Supplementary Figure 8. Scanning electron microscopy (SEM) images of (a) pristine copper foil and (b and c) PDADMA/Cu (PDADMA loading: 0.75 mg cm^{-2}). All scale bars are 10 μm.

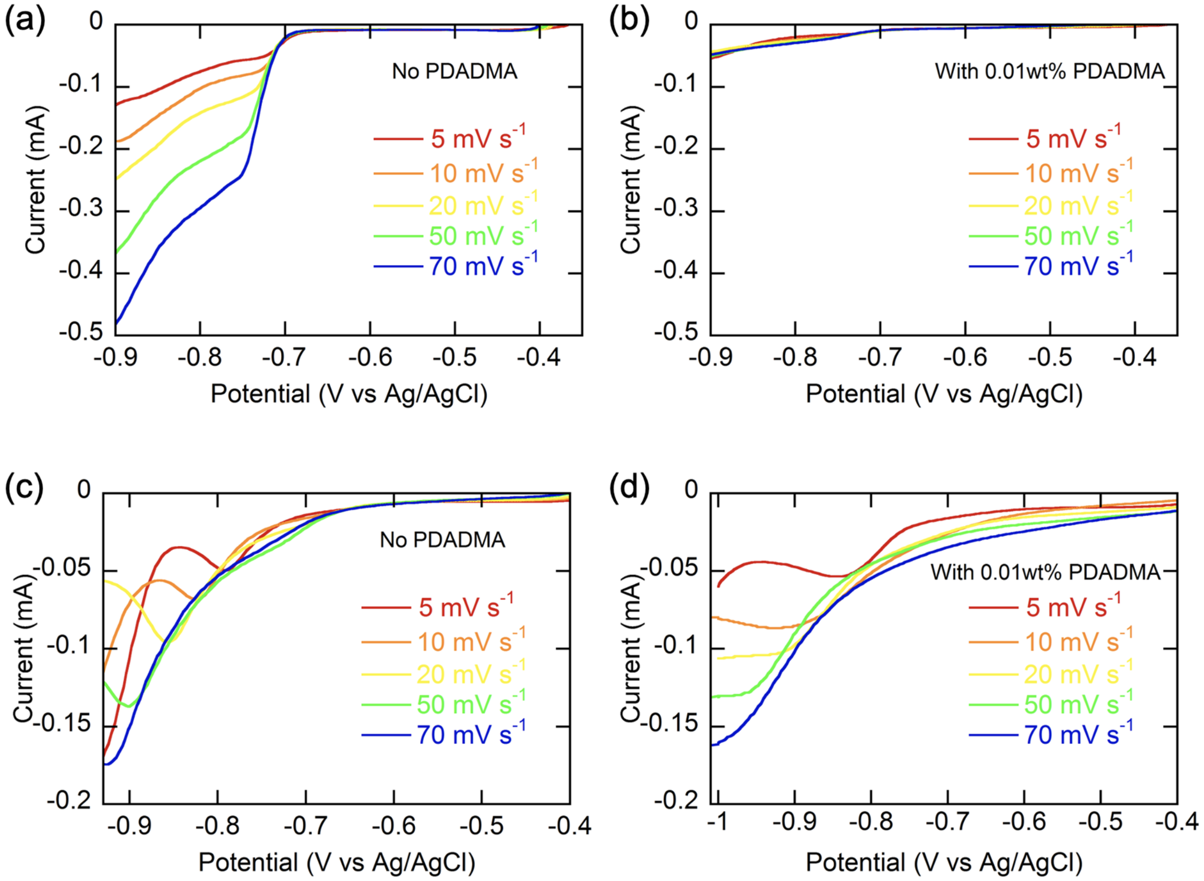

Supplementary Figure 9. (a-b) Linear sweep voltammograms of pristine copper foils during cathodic sweep in 10 mM Co(II) in 10 M LiCl (a) in the absence and (b) in the presence of 0.01wt% PDADMA as an additive in the electrolyte. (c-d) Linear sweep voltammograms of pristine copper foils during cathodic sweep in 10 mM Ni(II) in 10 M LiCl (a) in the absence and (b) in the presence of 0.01wt% PDADMA as an additive in the electrolyte. The scan rate was 5 mV s^{-1} and there was no stirring.

Calculation of diffusion coefficients from Supplementary Figure 9. Diffusion coefficients were calculated using the linear sweep voltammograms obtained in **Supplementary Figure 9**. The following equation for soluble-insoluble redox pairs was employed[3, 4]:

$$i_{\mathrm{p}} = 1.082nFAC\sqrt{\frac{nFDv}{RT\pi}}$$

where i_p is the peak current (A), n is the number of electrons transferred, C is the concentration of metal (mol cm^{-3}), A is the electrode area (cm^2), F is Faraday constant (96485 C mol^{-1}), υ is the scan rate (V s^{-1}), and D is the diffusion coefficient (cm^2 s^{-1}). Note that the current magnitude in linear sweep voltammetry contains partial contribution from metal deposition and also from hydrogen evolution. Therefore, the true partial contribution of metal deposition during linear sweep voltammogram was corrected by using Faradaic efficiency during the sweep processes, as determined in **Supplementary Figure 4**.

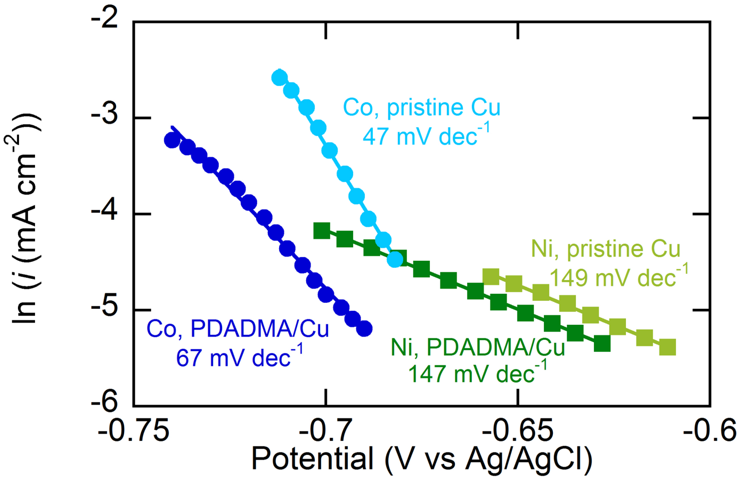

Supplementary Figure 10. Tafel plots of a single metal salt of 10 mM Co(II) or Ni(II) in 10 M LiCl using pristine Cu and PDADMA-loaded Cu (PDADMA loading: 0.75 mg cm^{-2}). Note that the current magnitude in voltammetry contains partial contribution from metal deposition and also from hydrogen evolution, which are indistinguishable. Therefore, the true partial contribution of metal deposition during the Tafel analysis was corrected by using average Faradaic efficiency values in near onset potential range (-0.7 to -0.6 V vs Ag/AgCl for Ni(II) and -0.8 V to -0.7 V vs Ag/AgCl for Co(II)), as determined in **Supplementary Figure 4**.

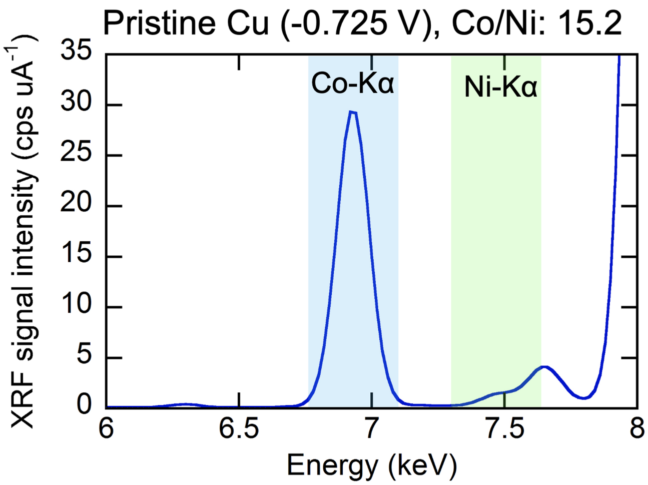

Supplementary Figure 11. XRF spectrum of cobalt and nickel in the electrodeposit formed using pristine copper at -0.725 V vs Ag/AgCl in 100 mM Co(II)+Ni(II) in 10 M LiCl.

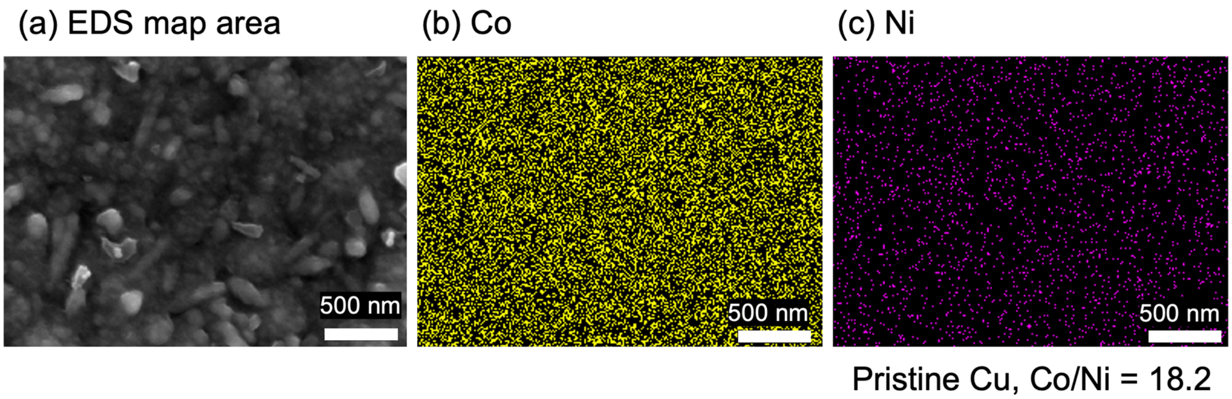

Supplementary Figure 12. EDS mapping of cobalt and nickel in the electrodeposit formed using pristine Cu at -0.725 V vs Ag/AgCl in 100 mM Co(II)+Ni(II) in 10 M LiCl. Scale bars for EDS and cobalt/nickel mapping are 500 nm.

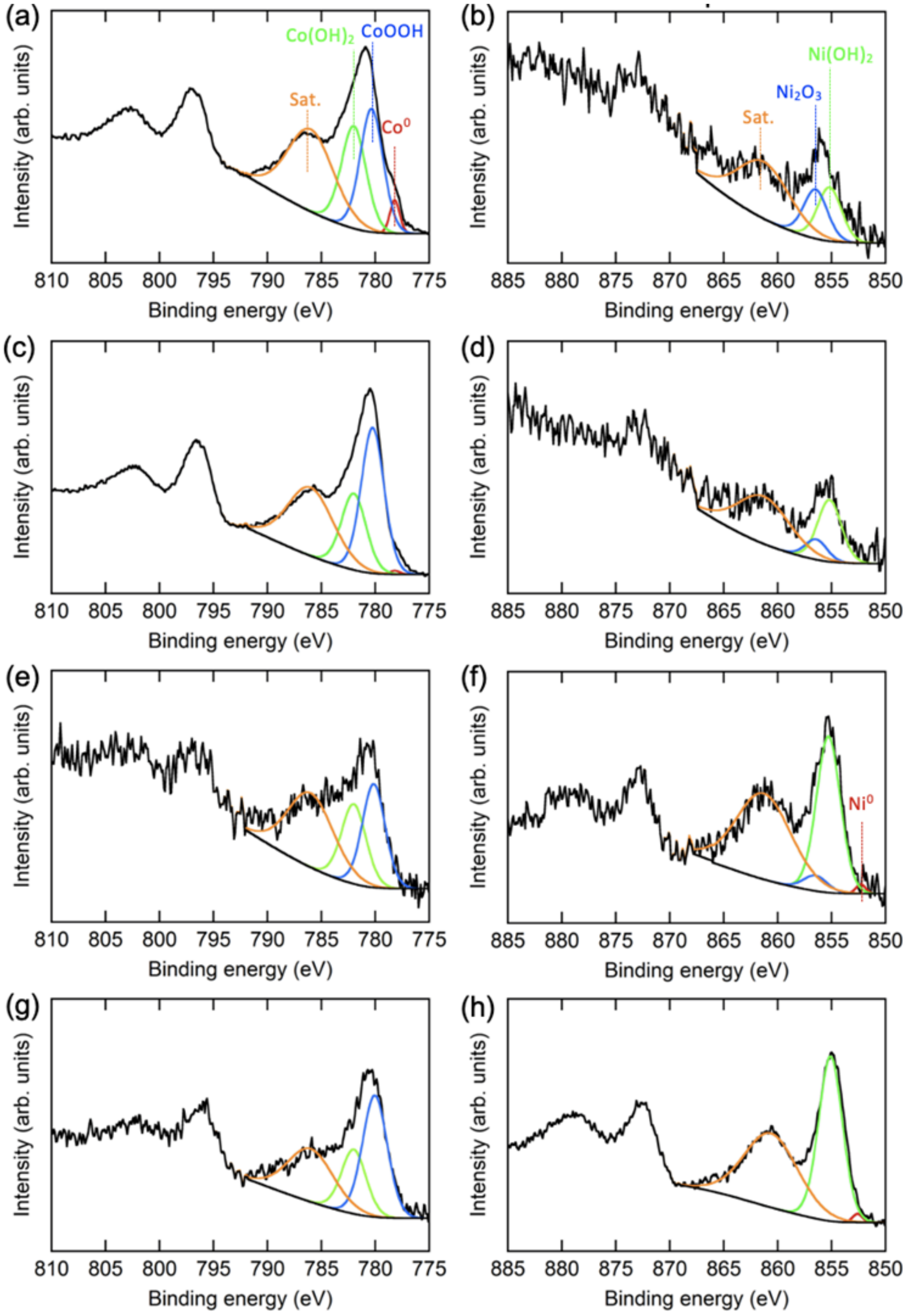

Supplementary Figure 13. High resolution Co2*p* and Ni2*p* spectra of the electrodeposits. (a) Co2*p* and (b) Ni2*p* spectrum after electrodeposition in 100 mM Co(II)+Ni(II) in 10 M LiCl at -0.725 V vs Ag/AgCl using pristine copper. (c) Co2*p* and (d) Ni2*p* spectrum after electrodeposition in 100 mM Co(II)+Ni(II) in 10 M LiCl at -0.725 V vs Ag/AgCl using PDADMA/Cu (PDADMA loading: 0.07 mg cm^{-2}). (e) Co2*p* and (f) Ni2*p* spectrum after electrodeposition in 10 mM Co(II)+Ni(II) in 10 M LiCl at -0.6 V vs Ag/AgCl using pristine copper. (g) Co2*p* and (h) Ni2*p* spectrum after electrodeposition in 10 mM Co(II)+Ni(II) in 10 M LiCl at -0.6 V vs Ag/AgCl using

PDADMA/Cu (PDADMA loading: 0.75 mg cm^{-2}). Parameters for curve-fitting of Co2*p* and Ni2*p* were determined from reported literature[5, 6, 7, 8].

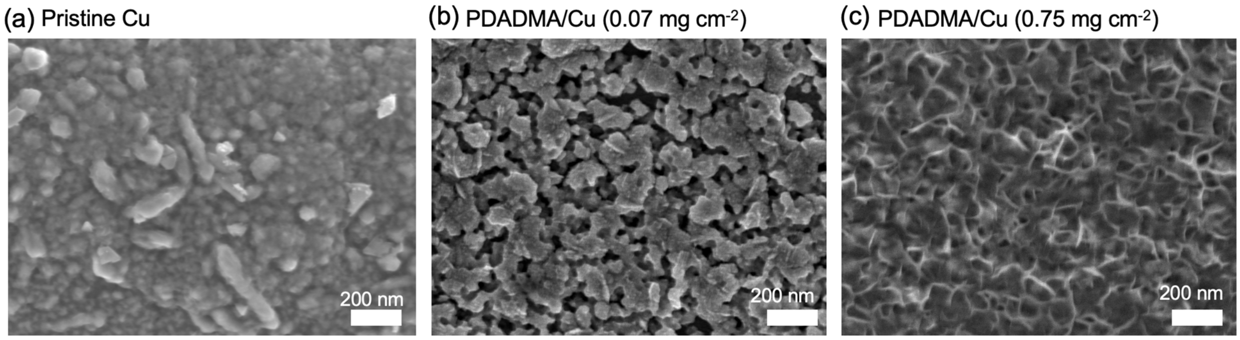

Supplementary Figure 14. Scanning electron microscopy (SEM) images of the cobalt and nickel electrodeposited on (a) pristine copper and (b) PDADMA/Cu (PDADMA loading: 0.07 mg cm^{-2}), and (c) PDADMA/Cu (PDADMA loading: 0.75 mg cm^{-2}) at -0.725 V vs Ag/AgCl in 100 mM Co(II)+Ni(II) in 10 M LiCl. Scale bars: 200 nm.

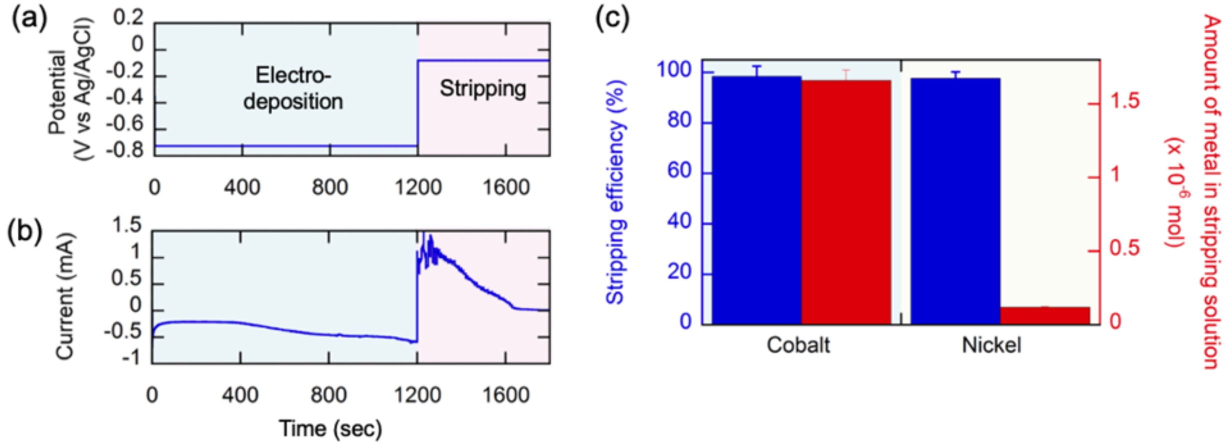

Supplementary Figure 15. Electrodeposition-stripping on PDADMA/Cu electrode (PDADMA loading: 0.07 mg cm^{-2}). (a) Potential and (b) current during the electrodeposition and stripping of cobalt and nickel. Electrodeposition was carried out at -0.725 V vs Ag/AgCl in 100 mM Co(II)+Ni(II) in 10 M LiCl and stripping was carried out at -0.08 V vs Ag/AgCl in 5 mM $NaNO_3$, whose pH was adjusted to 3 using HCl. (c) Stripping efficiency of the electrodeposited cobalt and nickel (left y-axis) and the amount of stripped cobalt and nickel (right y-axis) during stripping at -0.08 V vs Ag/AgCl. Error bars indicate standard error of the mean (n=2).

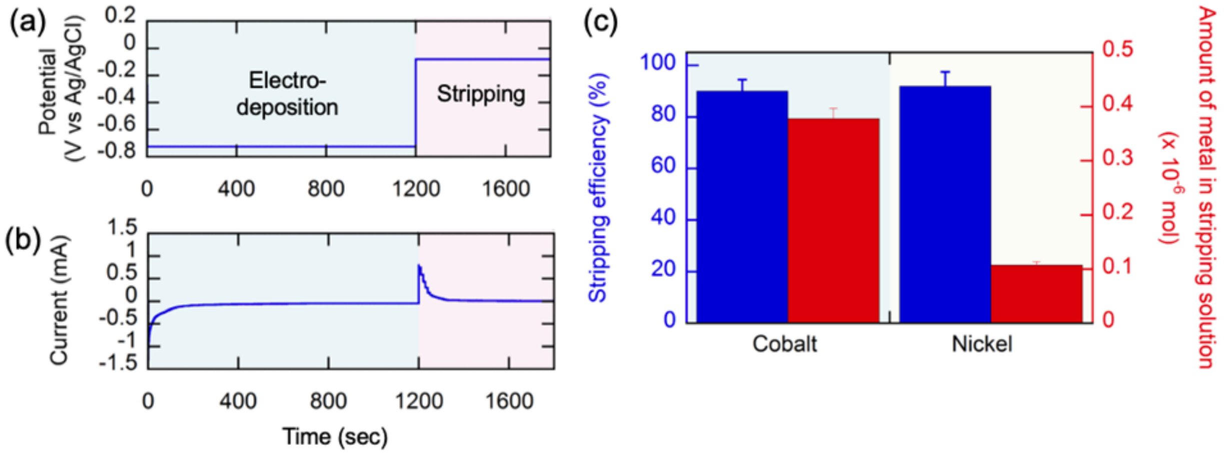

Supplementary Figure 16. Electrodeposition-stripping on PDADMA/Cu electrode (PDADMA loading: 0.75 mg cm^{-2}). (a) Potential and (b) current during the electrodeposition and stripping of cobalt and nickel. Electrodeposition was carried out at -0.725 V vs Ag/AgCl in 100 mM Co(II)+Ni(II) in 10 M LiCl and stripping was carried out at -0.08 V vs Ag/AgCl in 5 mM $NaNO_3$, whose pH was adjusted to 3 using HCl. (c) Stripping efficiency of the electrodeposited cobalt and nickel (left y-axis) and the amount of stripped cobalt and nickel (right y-axis) during stripping at -0.08 V vs Ag/AgCl. Error bars indicate standard error of the mean (n=2).

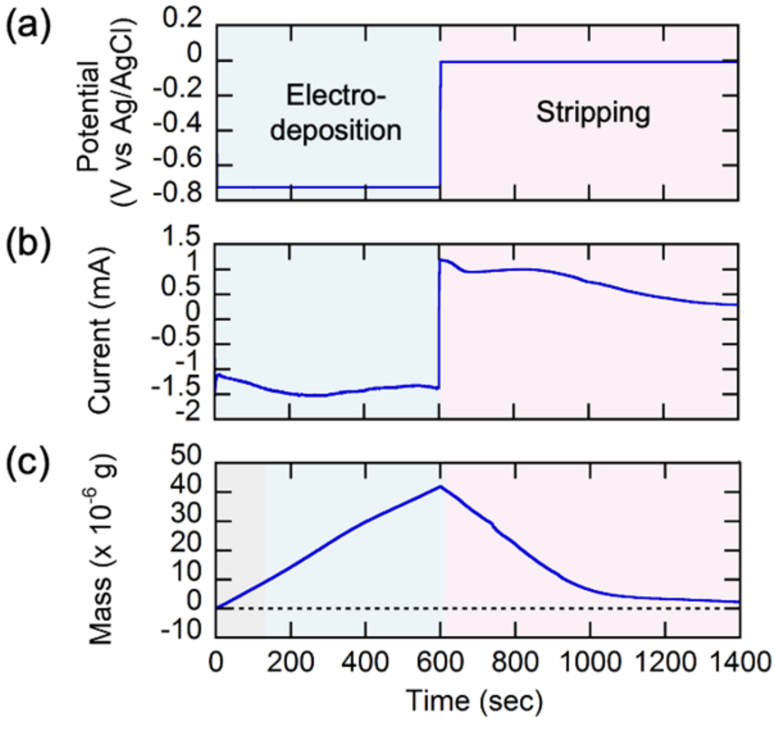

Supplementary Figure 17. EQCM study of electrodeposition-stripping on a Cu-coated quartz crystal. (a) Potential, (b) current, and (c) change in mass during the electrodeposition and stripping of cobalt and nickel. Electrodeposition was carried out at -0.725 V vs Ag/AgCl in 100

mM Co(II)+Ni(II) in 10 M LiCl and stripping was carried out at -0.08 V vs Ag/AgCl in 5 mM $NaNO_3$, whose pH was adjusted to 3 using HCl.

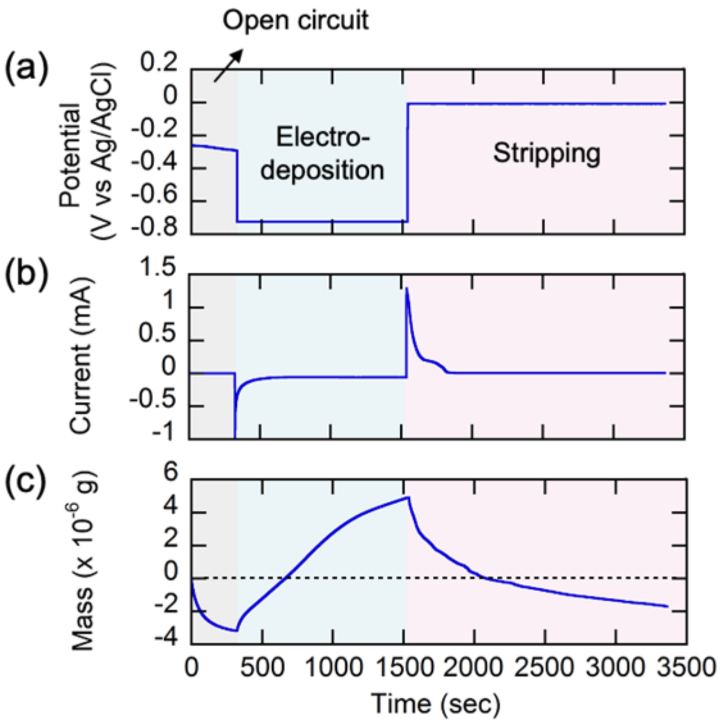

Supplementary Figure 18. EQCM study of electrodeposition-stripping on a PDADMA/Cu-coated quartz crystal (PDADMA loading: 0.75 mg cm^{-2}). (a) Potential, (b) current, and (c) change in mass during the electrodeposition and stripping of cobalt and nickel. Electrodeposition was carried out at -0.725 V vs Ag/AgCl in 100 mM Co(II)+Ni(II) in 10 M LiCl and stripping was carried out at -0.08 V vs Ag/AgCl in 5 mM $NaNO_3$, whose pH was adjusted to 3 using HCl.

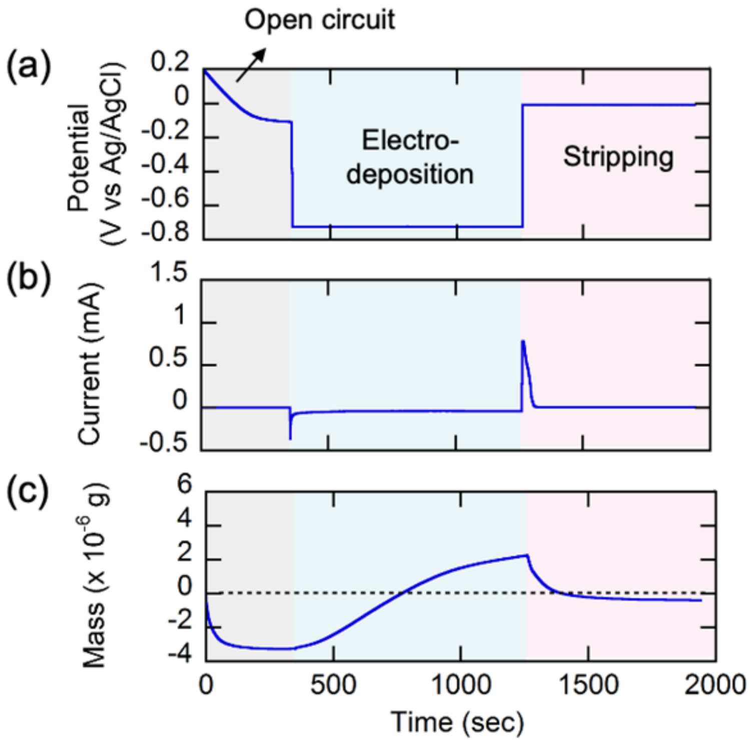

Supplementary Figure 19. EQCM study of electrodeposition-stripping on a PDADMA/Au-coated quartz crystal (PDADMA loading: 0.75 mg cm^{-2}). (a) Potential, (b) current, and (c) change in mass during the electrodeposition and stripping of cobalt and nickel. Electrodeposition was carried out at -0.725 V vs Ag/AgCl in 100 mM Co(II)+Ni(II) in 10 M LiCl and stripping was carried out at -0.08 V vs Ag/AgCl in 5 mM $NaNO_3$, whose pH was adjusted to 3 using HCl.

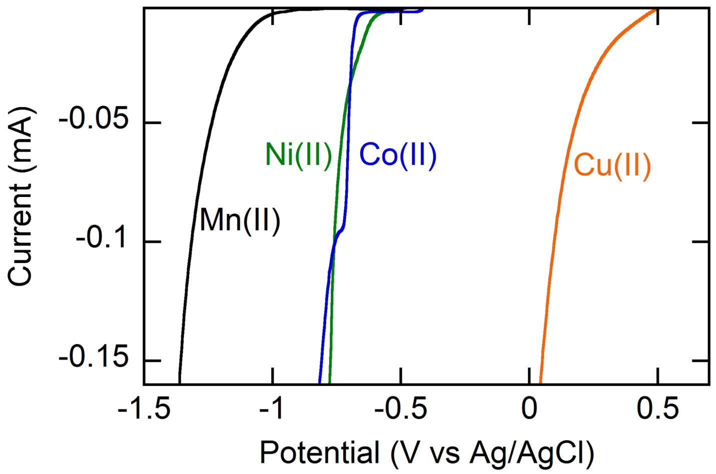

Supplementary Figure 20. Linear sweep voltammograms of a single metal salt of 10 mM Cu(II), Co(II), Ni(II) or Mn(II) in 10 M LiCl.

Technoeconomic analysis (TEA)

For the technoeconomic analysis, we put our basis on the experimental samplings/analysis results using practical Li-ion cells. The technoeconomic analysis was carried out under our experimental scales (e.g., electrolyte volume, working area of the electrode) for a given amount of spent LIBs cathode powder (which was 4 g in our study), then the cost and revenue were normalized to per kg basis. Even though this technique seems to be easily scalable, the use of a batch experimental scale allows for us to be conservative in calculating the energy/material consumptions. Electricity cost was assumed to be \$0.069/kWh[9]. The working area of the 2-D electrode was 0.5 cm^2. The market prices of various industrial-grade reagents were obtained from research papers, reports, and websites. Also, we assumed 95% recovery of cobalt and nickel. **Supplementary Figure 21** depicts the material and energy flow in the proposed process when recovering 4 g of NMC powder. The revenue and cost were normalized per kilogram basis.

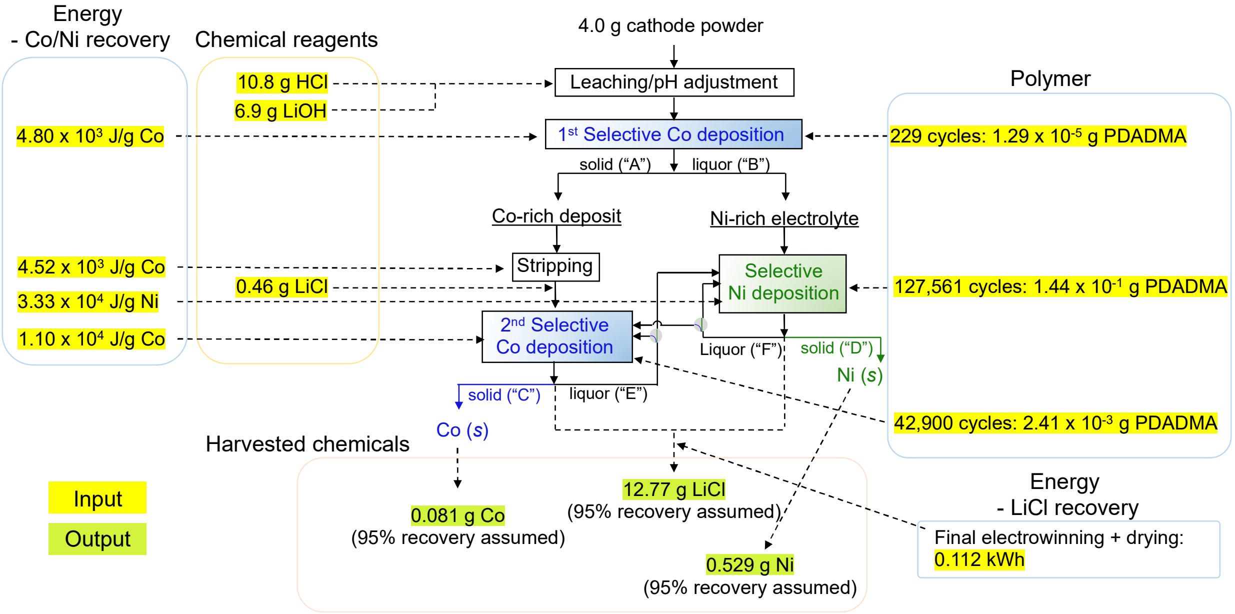

Supplementary Figure 21. Flow of the energy consumption and chemical reagents during the electrochemical recovery of cobalt and nickel proposed in this study.

(1) Leaching

(a) Material cost

- 30 mL of 10 M HCl (*10.8 g*) was used to leach *4 g* of NMC powder harvested from cylindrical Li-ion cells.
- After leaching, *6.9 g* LiOH was added to adjust the pH to 3.0.
- The market prices of HCl (\$0.15 kg^{-1}) and LiOH (\$12.18 kg^{-1}) were obtained from database reported in the EverBatt model developed by Arogonne National Laboratory[9].

(2) 1st selective Co deposition/stripping

(a) Material cost

- In our unit operation, *3.54×10^-4 g of cobalt* was deposited with the PDADMA loading of *1.88×10^-5 g*, so the normalized deposition capacity was found to be *18.83 g Co g^-1 PDADMA*.
- *229 cycles* of the unit operation are required for the recovery of 95% cobalt in 4 g NMC powder. We assumed, based on our EQCM analysis (**Supplementary Figure 18 and 19**), that each cycle of electrodeposition/stripping results in 0.3 % mass loss in PDADMA polymer (even though the loss can be minimized even more by controlling the duration of the stripping) – which translates into *1.29×10^-5 g PDADMA* loss for treating 4 g NMC powder in this stage. The market price of PDADMA was assumed to be \$2.5 kg^{-1}.[10]
- After repeated deposition/stripping cycles, *0.46 g of LiCl* was added in the stripping electrolyte to have desirable speciation of cobalt.
(b) Energy cost:
- From the unit operation, *1.7 J and 1.6 J* of electrical energies were consumed for the 1st electrodeposition and stripping of 3.54×10^{-4} *g* of cobalt, which translates to $4.80\times10^{3}\ J\ g^{-1}\ Co$ and $4.52\times10^{3}\ J\ g^{-1}\ Co$, respectively.

(3) 2nd selective Co deposition
(a) Material cost and revenue
- The unit operation results revealed that cobalt could be obtained with the normalized deposition capacity of *0.10 g Co/g PDADMA*. The final cobalt recovery was estimated to be *0.081 g*.
- Also, it turned out that *42,900 cycles* of the unit operations are required for 95% recovery of cobalt in 4 g NMC powder. The corresponding PDADMA loss accounts for 2.41×10^{-3} *g* PDADMA.
- We employed the market price of cobalt (\$51.33 kg^{-1}) reported in EverBatt model developed by Arogonne National Laboratory[9].
b) Energy cost:
- From the unit operation, $1.10\times10^{4}\ J\ g^{-1}\ Co$ of the electrical energy was consumed for the electrodeposition of cobalt with the purity > 96%.

(4) Selective Ni deposition
(a) Material cost and revenue
- The unit operation results revealed that nickel could be obtained with the normalized deposition capacity of *0.01 g Ni* g^{-1} *PDADMA*. The final nickel recovery was estimated to be *0.529 g*.
- Also, it turned out that *127,561 cycles* of the unit operation are required for 95% recovery of nickel in 4 g NMC powder. The corresponding PDADMA loss accounts for 1.44×10^{-1} *g PDADMA*.
- In the calculation of the revenue from nickel recovery, we employed the market price of nickel (\$11.30 kg^{-1}) reported in EverBatt model[9].
(b) Energy cost:
- From the unit operation, $3.33\times10^{4}\ J\ g^{-1}\ Ni$ of the electrical energy was consumed for the electrodeposition of nickel with the purity > 94%.

(5) Drying of the electrolyte and recovery of LiCl
(a) Material revenue
- We assumed the recovery rate of 95% lithium salt in the form of LiCl (*12.768 g*).
- The market price of LiCl (\$6.89 kg^{-1}) was converted from Li_2CO_3 cost based on lithium content[9].

(b) Energy cost

- After cobalt/nickel are recovered, the remaining transition metals are further removed by final bulk electrodeposition. In our experiment, the electrical energy consumed during the final electrodeposition was *0.022 kWh*.
- The resultant electrolyte was dried at 150 °C for 30 min in an oven to crystallize LiCl. Based on the technical specification of the oven, the energy consumption at 150 °C is 180 Wh h^{-1}, which translates to *0.09 kWh.* In this calculation, we were not able to efficiently utilize the electrical energy consumed by an oven because of the small scale of the electrolyte dried and LiCl crystallized (only 30 ml of electrolyte was put in a 50 L oven); if the drying process is optimized in a larger scale, the normalized energy consumption required for the crystallization of a given amount of LiCl would be significantly minimized.

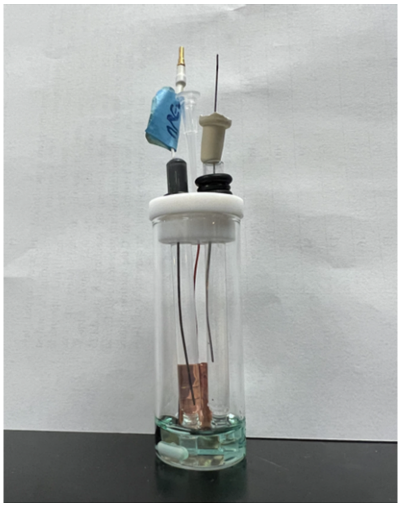

Supplementary Figure 22. A photographic picture of the electrochemical cell used in this study.

Supplementary Table 1. Comparison of various state-of-the-art techniques for separation of cobalt and nickel based on selectivity performance metrics.

Technique	Co, Ni concentration	Leaching solution/ background electrolyte	Key materials (precipitant/extractant /adsorbent/electrode)	Selectivity performance metric	Refs
Precipitation	[Co]: 9.05 g L^{-1}<br>[Ni]: 4.34 g L^{-1}<br>(after Mn recovery)	3 M H_2SO_4 +<br>3 vol% H_2O_2	Ni: $C_4H_8N_2O_2$<br>Co: NaOH	100 Ni over Co separation factor[a] (~48 Ni over Co on precipitate)	11
Solvent extraction	[Co]: 15 g L^{-1}<br>[Ni]: 21 g L^{-1}	4-8 M HCl	$[P_{8888}]$[oleate]	30,000 Co over Ni separation factor[b]	12
Solvent extraction	[Co]: 14 g L^{-1}<br>[Ni]: 15 g L^{-1}	2 M H_2SO_4 +<br>6 vol% H_2O_2	Cyanex 272	750 Co over Ni separation factor[b]	13
Adsorption	[Co]: 2.10 ppm<br>[Ni]: 1.98 ppm	4 M H_2SO_4 +<br>30wt% H_2O_2	(E)-4-[(2-mercaptophenyl)diazenyl] -2-nitrosonaphthalen-1-ol in γ-Al_2O_3 monoliths	62.7 Co over Ni separation factor[c]	14
Adsorption	[Co]: 2.10 ppm<br>[Ni]: 10.1 ppm	4 M H_2SO_4 +<br>30wt% H_2O_2	[(*E*)-4-((3-amino-4-hydroxyphenyl)diazenyl)naphthalen-1-ol (AHPDN)] in platelets of ZnO	17.1 Co over Ni separation factor[c]	15
Adsorption	[Co]: 5 μg mL^{-1}<br>[Ni]: 5 μg mL^{-1}	-	Ni(II)-imprinted amino-functionalized silica gel	280.03 Ni over Co selectivity coefficient[d]	16
Adsorption	[Co]: 1 μg mL^{-1}<br>[Ni]: 1 μg mL^{-1}	-	Ni(II) ion-imprinted polymer	14.1 Ni over Co selectivity coefficient[d]	17
Adsorption	[Co]: 10 mg L^{-1}<br>[Ni]: 10 mg L^{-1}	-	$Bi_2O(H_2O)_2(C_{14}H_2O_8)\cdot nH_2O$ MOF (SU-101)	Not available	18
Intercalation electrode membrane	[Co]: 0.1 M<br>[Ni]: 0.1 M	-	Mo_6S_8 (Chevrel phase) electrochemical transfer junction	99% Co over Ni selectivity factor[e]	19
Electrodialysis	[Co]: 0.01 M<br>[Ni]: 0.01 M	3–6 M HCl solution	Liquid membrane (trialkylbenzylammonium chloride + tri-n-octylamine in 1,2-dichloroethane)	145 Co over Ni separation factor[f]	20
Electrodeposition	[Co]: 0.1 M<br>[Ni]: 0.1 M	10 M LiCl	Poly(diallyldimethylammonium chloride) on copper	16.73 Co over Ni separation factor[c]	This study

[a] Separation factor: $(A/B)_{precipitate}/(A/B)_{initial\ solution\ concentration}$

[b] Separation factor is defined as D_{Co}/D_{Ni}, where D_{metal} is distribution coefficient of a metal in the extraction process.

[c] Separation factor: $(A/B)_{adsorbed\ or\ deposited}/(A/B)_{initial\ solution\ concentration}$

[d] Selectivity coefficient: (D_{Ni}/D_{Co}), where $D=Q/C_e$ (Q: adsorption capacity in mg g^{-1}, C_e: equilibrium concentration)

[e] Selectivity factor: ratio n(*Co*)/(n(*Co*)+ n(*Ni*)), where n is the number of moles in the recovery compartment.

[f] Separation factor: $(A/B)_{in\ strip\ solution}/(A/B)_{in\ feed\ solution}$

Supplementary Table 2. The cost/benefit from chemical reagents to treat 4 g of NMC powders by the present electrochemical recovery process.

Substance	Quantity (g)	Price ($ kg^{-1})	Benefits ($)	Benefits per kg ($ kg^{-1})	Ref
HCl	10.800	0.150	-0.002	-0.405	[9]
LiOH	6.900	12.180	-0.084	-21.011	[9]
PDADMA-1st deposition/stripping	1.29E-05	2.500	-3.23E-08	-8.06E-06	[10]
LiCl	0.460	6.890	-0.003	-0.792	[9]
PDADMA-selective cobalt deposition	0.002	2.500	-6.03E-06	-0.002	[10]
PDADMA-selective nickel deposition	0.144	2.500	-3.60E-04	-0.090	[10]
Cobalt	0.081	51.330	0.004	1.040	[9]
Nickel	0.529	11.300	0.006	1.495	[9]
Harvested LiCl	12.768	6.890[a]	0.088	21.993	[9]
Sum			0.009	2.230	

[a] The market price of LiCl ($6.89 kg^{-1}) was converted from Li_2CO_3 cost based on lithium content[9]

Supplementary Table 3. The cost from the energy consumption to treat 4 g of NMC powders by the present electrochemical recovery process.

Stage	Energy (J g^{-1})	Energy (kWh)	Cost ($)	Cost per kg ($ kg^{-1})
1st cobalt deposition	4800	0.00011	7.46E-06	0.0019
1st cobalt stripping	4520	0.00010	7.024E-06	0.0018
Selective cobalt deposition	11000	0.00025	1.709E-05	0.0043
Selective nickel deposition	33300	0.00490	0.0003379	0.0845
Bulk electrodeposition before drying		0.02217	0.0015297	0.3824
Drying		0.09	0.00621	1.5525
Sum		0.1175	0.0081	2.0273

Supplementary References

1. Hansal WEG, Tury B, Halmdienst M, Varsányi ML, Kautek W. Pulse reverse plating of Ni–Co alloys: Deposition kinetics of Watts, sulfamate and chloride electrolytes. *Electrochimica Acta* **52**, 1145-1151 (2006).

2. Karimzadeh A, Aliofkhazraei M, Walsh FC. A review of electrodeposited Ni-Co alloy and composite coatings: Microstructure, properties and applications. *Surface and Coatings Technology* **372**, 463-498 (2019).

3. Yoon S, Choi S. Spectroelectrochemical Behavior of Cr, Fe, Co, and Ni in LiCl-KCl Molten Salt for Decontaminating Radioactive Metallic Wastes. *Journal of the Electrochemical Society* **168**, 013504 (2021).

4. Lane GH, Best AS, MacFarlane DR, Forsyth M, Bayley PM, Hollenkamp AF. The electrochemistry of lithium in ionic liquid/organic diluent mixtures. *Electrochimica Acta* **55**, 8947-8952 (2010).

5. Dong D*, et al.* Electrochromic properties of NiOx:H films deposited by DC magnetron sputtering for ITO/NiOx:H/ZrO2/WO3/ITO device. *Applied Surface Science* **357**, 799-805 (2015).

6. Ali-Löytty H*, et al.* Ambient-Pressure XPS Study of a Ni–Fe Electrocatalyst for the Oxygen Evolution Reaction. *The Journal of Physical Chemistry C* **120**, 2247-2253 (2016).

7. Ristova MM, Francis C, Toma FM, Yu KM, Walukiewicz W. Electrochemical modification of the optical and electrical properties of Cd-rich NixCd1–xO alloys. *Solar Energy Materials and Solar Cells* **147**, 127-133 (2016).

8. Han Y*, et al.* Observing the Electrochemical Oxidation of Co Metal at the Solid/Liquid Interface Using Ambient Pressure X-ray Photoelectron Spectroscopy. *The Journal of Physical Chemistry B* **122**, 666-671 (2018).

9. Dai Q, Spangenberger J, Ahmed S, Gaines L, Kelly JC, Wang M. EverBatt: A Closed-loop Battery Recycling Cost and Environmental Impacts Model (ANL-19/16). *Argonne National Laboratory, Energy Systems Division*, (2019).

10. https://bluwat01.en.made-in-china.com/product/YvJErxnFFiWm/China-Polydadmac-Powder-poly-diallyl-dimethyl-ammonium-chloride-.html
(Website visiting date: 08/07/2021).

11. Yang X, Zhang Y, Meng Q, Dong P, Ning P, Li Q. Recovery of valuable metals from mixed spent lithium-ion batteries by multi-step directional precipitation. *RSC Advances* **11**, 268-277 (2021).

12. Othman EA, van der Ham AGJ, Miedema H, Kersten SRA. Recovery of metals from spent lithium-ion batteries using ionic liquid [P8888][Oleate]. *Separation and Purification Technology* **252**, 117435 (2020).

13. Kang J, Senanayake G, Sohn J, Shin SM. Recovery of cobalt sulfate from spent lithium ion batteries by reductive leaching and solvent extraction with Cyanex 272. *Hydrometallurgy* **100**, 168-171 (2010).

14. Gomaa H, Shenashen MA, Yamaguchi H, Alamoudi AS, El-Safty SA. Extraction and recovery of Co2+ ions from spent lithium-ion batteries using hierarchical mesosponge γ-Al2O3 monolith extractors. *Green Chemistry* **20**, 1841-1857 (2018).

15. Gomaa H*, et al.* Three-Dimensional, Vertical Platelets of ZnO Carriers for Selective Extraction of Cobalt Ions from Waste Printed Circuit Boards. *ACS Sustainable Chemistry & Engineering* **6**, 13813-13825 (2018).

16. Jiang N, Chang X, Zheng H, He Q, Hu Z. Selective solid-phase extraction of nickel(II) using a surface-imprinted silica gel sorbent. *Analytica Chimica Acta* **577**, 225-231 (2006).

17. Abbasi S, Roushani M, Khani H, Sahraei R, Mansouri G. Synthesis and application of ion-imprinted polymer nanoparticles for the determination of nickel ions. *Spectrochimica Acta Part A: Molecular and Biomolecular Spectroscopy* **140**, 534-543 (2015).

18. Piątek J*, et al.* Toward Sustainable Li-Ion Battery Recycling: Green Metal–Organic Framework as a Molecular Sieve for the Selective Separation of Cobalt and Nickel. *ACS Sustainable Chemistry & Engineering* **9**, 9770-9778 (2021).

19. Guyot E*, et al.* Mo6S8Electrochemical Transfer Junction for Selective Extraction of Co2+-Ions from Their Mixture with Ni2+-Ions. *Journal of The Electrochemical Society* **160**, A420-A425 (2013).

20. Sadyrbaeva TZ. Separation of cobalt(II) from nickel(II) by a hybrid liquid membrane–electrodialysis process using anion exchange carriers. *Desalination* **365**, 167-175 (2015).
